# Evaluating a designed vocational rehabilitation program based on society ecosystems theory for young and middle-aged patients with coronary heart disease: a randomized controlled trial

**DOI:** 10.3389/fpubh.2026.1674523

**Published:** 2026-01-26

**Authors:** Yaoyao Hu, Qi Zhang, Taihua Zhou, Yuling Yang, Xiaorui Feng, Xingxing Ji, Lihua Zhang, Lingyun Zhu

**Affiliations:** 1 Affiliated Hospital of Jiangnan University, Wuxi, China; 2 School of Nursing, Suzhou Medical College of Soochow University, Suzhou, China; 3 Rehabilitation Department of Northern Jiangsu People’s Hospital, Yangzhou, Jiangsu, China

**Keywords:** anxiety, coronary heart disease, quality of life, randomized controlled trial, return to work, return to work self-efficacy, vocational rehabilitation

## Abstract

**Background:**

Coronary heart disease (CHD) is increasingly affecting younger adults, but many face difficulties returning to work, exacerbating financial burdens. Although vocational rehabilitation may improve employment outcomes, evidence for Chinese CHD patients remains scarce. Therefore, the goal of this study is to improve the employment status of middle-aged and young patients with CHD by implementing vocational rehabilitation measures based on society ecosystems theory.

**Methods:**

The vocational rehabilitation program was developed in a multi-stage process. First, a preliminary draft was formulated based on a literature review, expert panels, and semi-structured interviews. Subsequently, this draft was refined and validated through a two-round Delphi expert consensus process. Following this, a pilot study was conducted to assess its feasibility. Finally, we evaluated the finalized program in a single-center randomized controlled trial, where 158 participants were equally randomized to receive either the 3-month intervention (*n* = 79) or usual care (*n* = 79). Return-to-work rates were the primary outcome indicator, with anxiety, quality of life, and return-to-work self-efficacy as secondary outcome indicators.

**Results:**

Following the 3-month intervention, the vocational rehabilitation intervention significantly improved return-to-work rates compared to the control group (87.1% vs. 61.6%; *χ*^2^ = 12.114, *p* < 0.001). In addition, return-to-work self-efficacy, quality of life, and anxiety improved in the intervention group after the intervention, and the difference was statistically significant compared to the control group (all *p* < 0.05). After adjusting for demographic characteristics and clinically relevant factors, the logistic regression model results robustly demonstrated that patients in the intervention group had 5.204 times the odds of successfully returning to work compared to those in the control group [95% CI (2.060, 13.148), *p* < 0.001].

**Conclusion:**

Our findings indicate that ecosystem theory-based vocational rehabilitation can effectively enhance employment outcomes in young and middle-aged patients with CHD. Healthcare professionals should consider multidimensional factors when developing return-to-work interventions, with future efforts incorporating workplace-related considerations.

## 1 Background

Coronary heart disease (CHD) is characterized by high incidence, high mortality, and a trend toward affecting younger populations (1, 2). While percutaneous coronary intervention (PCI) enables shorter hospital stays, many young and middle-aged patients in China intend to return to work (RTW) quickly post-discharge (3). However, with the promotion of China’s delayed retirement policy, the working life of patients with CHD has been further extended, which has created new challenges for working-age patients.

Cardiac rehabilitation traditionally integrates multiple prescriptions (4), yet vocational rehabilitation (VR) remains underemphasized. While European data indicate high RTW rates (76–89%) (5, 6). This stands in stark contrast to data from China, where merely 55.9% of PCI patients had returned to work within 1 year (7), and our survey found only 61.4% did so within 3 months (8). These low RTW rates correlate with reduced quality of life (QoL) and higher depression risk (4, 9), underscoring the need for structured VR.

VR effectively helps overcome employment barriers, with evidence showing it reduces RTW time and improves outcomes (10–12). However, it is rarely implemented in China. RTW is a complex process modulated by demographic, occupational, psychological, disease-related, and social factors (5, 13–19). The society ecosystems theory (SET), which examines interactions at the micro-, meso-, and macro-system levels, provides an ideal framework to conceptualize these influences (20, 21). This approach has been proven effective in chronic disease management (22, 23). To address this gap, this study developed a multilevel VR intervention guided by SET for young and middle-aged CHD patients. A randomized controlled trial (RCT) was designed to improve participants’ RTW rates, enhance their return-to-work self-efficacy (RTW-SE), reduce anxiety, and promote overall QoL.

## 2 Methods

### 2.1 Research hypothesis

We hypothesized that individuals receiving VR intervention have a higher RTW rate than those receiving conventional care, in addition to better RTW-SE and QoL and lower levels of anxiety than controls.

### 2.2 VR program development

The intervention was created through a multi-step, iterative process that made sure it was scientifically sound, clinically relevant, and possible.

#### 2.2.1 Comprehensive literature review

This section aimed to review the existing literature on VR in CHD patients, thereby establishing a foundation for the preliminary VR intervention developed in this study. A comprehensive literature search was performed across multiple electronic databases, including Web of Science Core Collection and PubMed for international literature, and China National Knowledge Infrastructure (CNKI) and Wanfang Data for Chinese literature, covering all records from their inception to April 2024. The search strategy employed topic terms related to “coronary heart disease” AND “vocational rehabilitation.” After screening, deduplication, and final review based on inclusion criteria (peer-reviewed journal articles focusing on VR) and exclusion criteria (conference papers, experiential summaries, etc.), 533 articles (495 English and 38 Chinese) were included. The analysis identified key factors influencing RTW in CHD patients, leading to the construction of a preliminary VR framework grounded in the SET, which categorized influencing factors and intervention strategies into micro-, meso-, and macro-systems. This framework was further refined through discussions within our multidisciplinary research team, which included cardiologists, physical and rehabilitation medicine specialists, nursing scientists, and clinical psychologists.

#### 2.2.2 Expert panels

The main focus of this phase was to integrate collective expertise and draft preliminary intervention plans and supporting materials. Two expert panel discussion sessions were convened, each lasting between 90 and 120 min. Each panel was composed of three clinical cardiologists, one physical and rehabilitation medicine specialist, one psychologist, three nursing specialists, and two nursing educators. The discussions focused on key intervention components, delivery methods, frequency, and potential barriers. A preliminary draft of the intervention was subsequently developed by synthesizing the insights and consensus achieved within the groups.

#### 2.2.3 Interviews with patients

To ground the intervention in real-world patient experiences and needs, we conducted semi-structured interviews with 16 CHD patients. The interview guide was developed based on study objectives and expert consultation, covering: (a) illness experiences from onset to treatment; (b) impact on work capacity and daily life; (c) thoughts and concerns about RTW; and (d) specific VR needs and desired support systems. Each interview lasted 30–60 min, was audio-recorded with informed consent, and transcribed verbatim for thematic analysis. The analysis yielded critical insights that reshaped the intervention:

1. Shift in Core Objective: From a primary focus on physical recovery to an equal emphasis on psychological reconstruction (addressing anxiety, fear of workplace exclusion, and rebuilding confidence).
2. Evolution of Strategy: From generic advice to personalized plans, including clear return-to-work pathways, skill training, and multidisciplinary support teams.
3. Expansion of Framework: From an individual focus to encompassing the entire social ecosystem, addressing the need for sustained psychosocial support and understanding from family, workplace, and society.

#### 2.2.4 Expert validation

A two-round Delphi Method was employed to refine the intervention protocol. A panel of 15 experts was purposively selected based on the following predefined criteria: (1) ≥ 10 years of work experience and an associate senior professional title or higher; (2) professional expertise in cardiology, clinical nursing, nursing management, rehabilitation medicine, or rehabilitation nursing; and (3) voluntary participation. The panel (aged 34–54, with 11–30 years of experience, all holding bachelor’s degrees or higher) was formed to ensure complementary expertise.

The questionnaires were distributed and collected via email. In the first round, the initial intervention protocol and materials were sent to the panel. Upon receipt of the responses, expert ratings and open-ended comments were systematically analyzed. This analysis informed an iterative refinement of the content, involving the deletion, modification, and addition of items, which constituted the second-round questionnaire. The second round followed an identical procedure for distribution and collection, with the returned feedback undergoing equally rigorous analysis. The consultations confirmed the protocol’s scientific validity, importance, and feasibility.

#### 2.2.5 Final refinement

A pilot study was conducted with a convenience sample of 10 eligible patients to assess the feasibility of the intervention and identify any practical issues. Participant feedback on content, duration, and usability led to key modifications: (1) Peer Support: Unstructured group discussions were replaced with sessions guided by structured core topic cards to maintain focus and prevent off-topic conversations. (2) Family Support: A detailed family support checklist was created, providing actionable recommendations (e.g., assisting with fatigue management, encouraging social participation) to translate family willingness into effective, non-intrusive support.

### 2.3 RCT design

**Figure 1:**
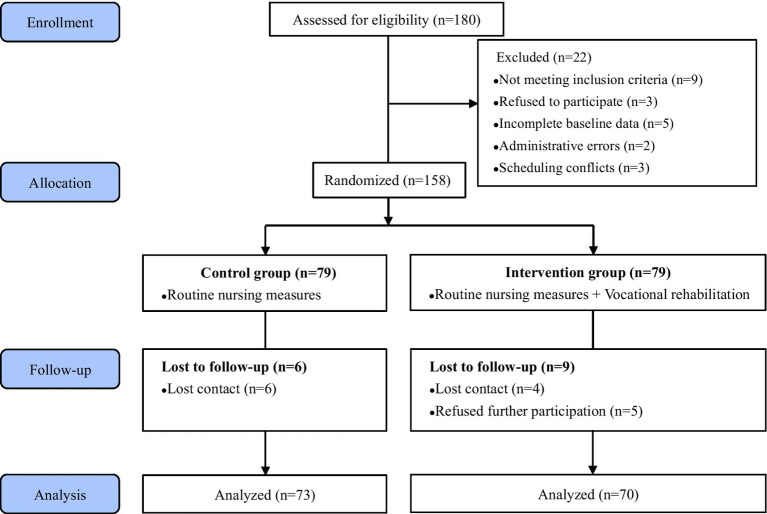
Study flowchart.

This was a RCT conducted from August 2024 to May 2025 at the Affiliated Hospital of Jiangnan University in Wuxi, Jiangsu Province. A total of 158 eligible patients were enrolled after providing informed consent and randomly assigned (1:1) to either the control group (routine nursing care) or the intervention group (routine care plus VR). Duration of the intervention was 3 months. This research was designed and implemented in compliance with the Consolidated Standards of Reporting Trials guidelines for randomized controlled trials Figure 1 presents the flow of this study.

### 2.4 Participants

#### 2.4.1 Inclusion and exclusion criteria

Inclusion criteria: (1) Age 18 to 65 (because the legal retirement age in our country is 65), (2) First diagnosis of CHD, (3) Paid employment at the time of diagnosis and not yet returned to work, (4) Able to communicate and interact normally, (5) Understand the own medical condition, (6) Able to use a smartphone, (7) Sign the paper version of the informed consent form. Exclusion criteria: (1) Combined with other diseases that affect survival time, (2) Will leave Wuxi within 3 months; (3) Are participating in other studies, (4) Have no plans to return to their jobs.

#### 2.4.2 Sample size

Based on a pivotal previous study of transitional care (24), which demonstrated a RTW rate of 90.5% (38/42) in the intervention group compared to 66.7% (28/42) in the control group (an absolute improvement of 23.8%), we estimated that our intervention would yield a clinically significant effect. To ensure a robust sample size that could detect a meaningful effect even if the efficacy of our intervention was somewhat lower than this reference, we conservatively set the hypothesized absolute improvement at 20 percentage points. Thus, we anticipated RTW rates of 81.4% in the intervention group and 61.4% in the control group, with the latter rate informed by other literature (7). Using G*Power software (version 3.1) to calculate the power for detecting a difference in proportions between two independent groups, with a significance level (*α*) of 0.05 and statistical power (1-*β*) of 80%, we determined that each group requires at least 63 participants. Accounting for a 20% attrition rate, we plan to recruit 79 participants per group, resulting in a total sample size of 158 participants.

### 2.5 Recruitment, allocation, randomization, and blinding

Participants were recruited from the cardiovascular medicine department, with trial advertisements posted in cardiology inpatient wards. Following a comprehensive study explanation by research staff, eligible participants provided written informed consent. In a 1:1 ratio, eligible participants were randomly allocated to the VR group (*n* = 79) or the control group (*n* = 79). The randomization sequences were generated using SPSS 29.0 software by an independent graduate nursing researcher, who had no further involvement in study implementation or data collection. The randomization assignments were saved in sealed and opaque envelopes, which were kept in a locked box under the custody of a designated person. During participant enrollment, the investigators opened these envelopes in sequential order according to the screening sequence and assigned participants to either the control group or the VR group based on the allocation information contained within. Due to the nature of the intervention, both participants and intervention providers were aware of group allocation. However, intervention group participants were instructed to maintain confidentiality regarding treatment details. The intervention team solely administered treatment and had no involvement in participant allocation, data collection, or analysis, with explicit instructions against disclosing allocation information. Outcome assessors and statisticians remained blinded throughout the study.

### 2.6 Intervention

#### 2.6.1 The control group

The control group received routine nursing measures. (1) During hospitalization: Patients received standardized education on CHD, covering risk factors, dietary strategies, weight management, physical activity recommendations, and blood pressure/lipid control protocols. Following each education session, participants were provided with either printed or digital versions of the educational manual. (2) For post-discharge follow-up: Designated nurses conducted regular follow-ups through WeChat (It is China’s dominant super-app integrating messaging, social media, and mobile payment functions, with over 1.3 billion monthly active users) or telephone consultations. These sessions involved delivering personalized recommendations regarding medication adherence, nutritional planning, exercise regimens, and scheduled re-examinations, while simultaneously addressing any rehabilitation-related queries from patients.

#### 2.6.2 Intervention group

**Figure 2:**
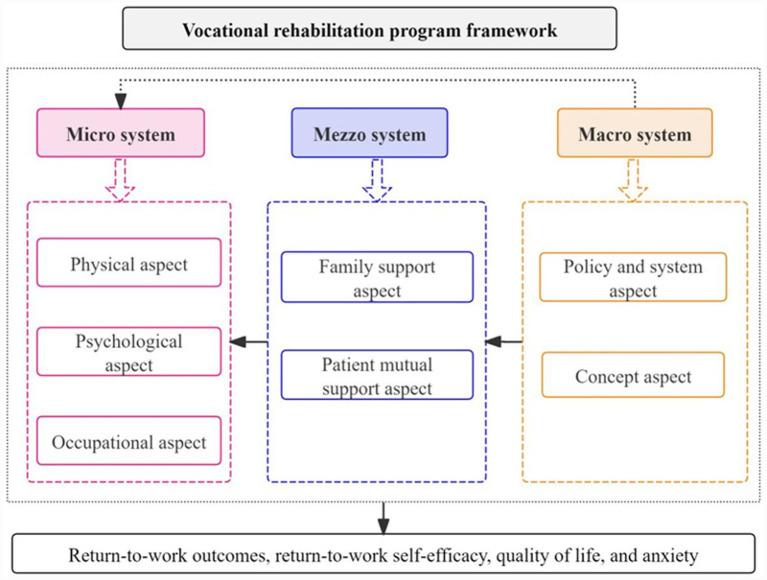
VR program framework.

The interventions addressed three system levels: (1) Micro system: Individual physical recovery, RTW-SE, and job adaptation. (2) Mezzo system: Family role adjustment and peer support. (3) Macro system: Policy navigation and disease cognition. The framework of VR program is given in Figure 2.

**Figure 3:**
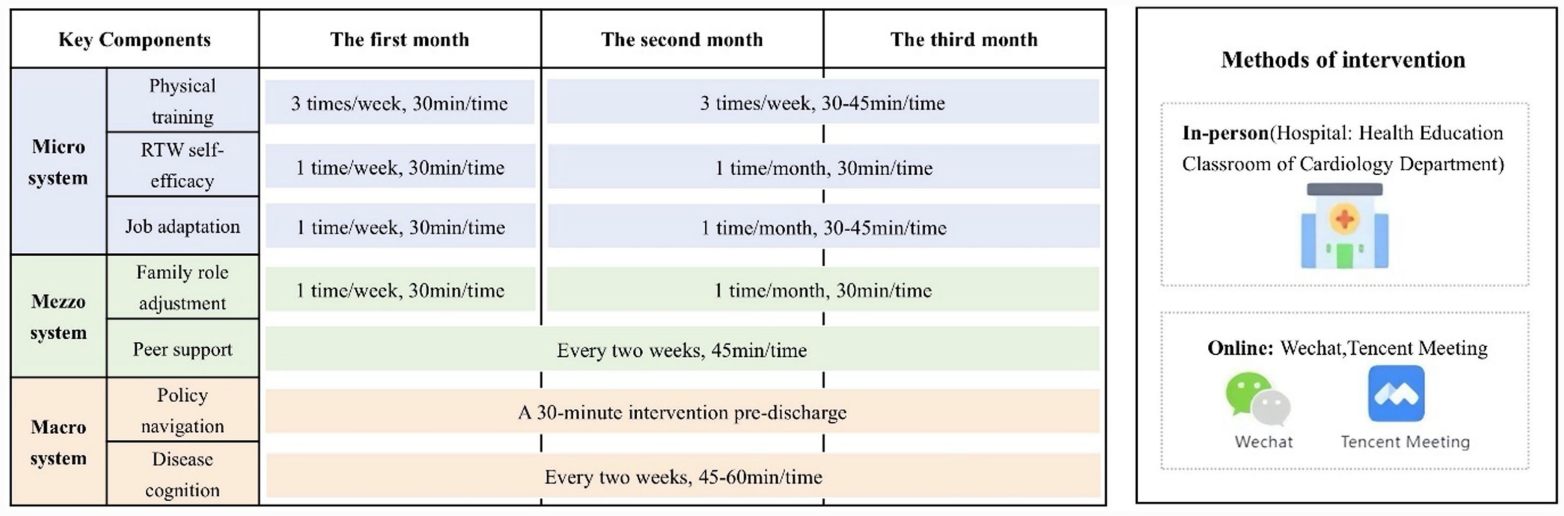
Delivery methods, schedule of the VR program (The implementation is detailed in Table 2).

The interventions were delivered using a combination of online and in-person formats. During hospitalization, the sessions were conducted in-person, then transitioned to online after discharge. All in-person sessions took place in the patient health education room of the cardiology ward. Online courses were delivered through WeChat or Tencent Meeting (a cloud-based video conferencing platform widely used in China, with over 300 million users, supporting high-definition audio/video calls, screen sharing, online collaboration, and other functions). The duration of the intervention was established at 3 months based on previous research (25, 26). The overview and detailed specifications of the VR program are presented in Tables 1, 2. The VR implementation timeline and procedures were shown in Figure 3.

**Table 1:** The outline content of VR.

SET	Intervention elements	Delivery mode	Objectives of the intervention
Micro system	Physical training	Mixed (in-person + online)	To optimize the physical health of patients
	RTW-SE		To enhance the self-efficacy of patients in their RTW process
	Job adaptation		Assist patients in adjusting to changes, balancing work demands, and adapting to their health conditions.
Mezzo system	Family role adjustment	Mixed (in-person + online)	Reallocate family roles and responsibilities to offer structured support to the patient.
	Peer support	Online	Give full play to peer power to promote patients’ VR
Macro system	Policy navigation	In-person	Help patients understand the policy system and reduce their financial burden
	Disease cognition	Mixed (in-person + online)	Ensure that patients have an accurate understanding of the disease

VR, Vocational rehabilitation; CHD, Coronary heart disease; RTW, Return to work; RTW-SE, Return to work self-efficacy.

**Table 2:** Content of occupational rehabilitation intervention.

SET elements	Aspects of intervention	Intervention content	Delivery
Micro system	Physical aspect	1. Goal: To optimize physical fitness and ensure safe exercise progression. 2. The protocol was periodized: Patients received risk-stratified, individualized exercise prescriptions. (1) Early stage (1st - 4th week of illness): Focused on low-intensity aerobic exercise (e.g., walking, Tai Chi) (2) Intermediate/Late Phase (Week 5 onward): Progressed to moderate-intensity aerobic exercise (e.g., brisk walking, swimming) and flexibility training (e.g., stretching, yoga).	In-person (hospital) transitioning to remote supervision (post-discharge). Implementing personnel: Cardiology nurses, physicians
	Psychological aspect	1. Goal: To enhance self-efficacy for RTW. 2. A multi-component intervention included: (1) Facilitated sessions to identify personal RTW barriers/enablers and set realistic goals; (2) Structured peer modeling through guided sharing from successful RTW patients; (3) Support journaling to document and reinforce perceived support from social networks. Patients were provided with a journal template prompting them to daily record: One instance of support received (from family, friends, colleagues, or healthcare providers). One positive action they took toward their RTW goal (e.g., “I walked for 20 min”). One positive thought or affirmation about their capability. Patients were instructed to review their journals weekly to visually consolidate evidence of their progress and social support, thereby strengthening their belief in their own capabilities and resources. (4) Send educational content (articles/videos) related to returning to work through WeChat groups or WeChat official accounts.	Delivery: In-person (hospital) transitioning to remote (post-discharge). Implementing personnel: Psychologists and nursing professionals
	Occupational aspect	1. Goal: To facilitate job adjustment and sustainable reintegration into the workforce. 2. Content: A multidisciplinary team (cardiologists, nurses, rehabilitators) conducted: (1) Assessment: The rehabilitator conducted a semi-structured interview to map the patient’s pre-illness job demands (physical, cognitive, psychosocial), current functional capacity, and perceived workplace barriers. The cardiologist provided medical clearance and specific activity restrictions. (2) Goal-Setting: In a collaborative session, the team and patient reviewed the assessment findings. Using the SMART framework (Specific, Measurable, Achievable, Relevant, Time-bound), they co-established primary vocational goals (e.g., “Return to a modified-duty desk role within 8 weeks”) (3) Development of a Graduated Return-to-Work Plan with Simulation: (a) Simulation Training: To bridge the gap between clinical recovery and work readiness, the rehabilitator facilitated simulated work tasks. For a patient with light physical demands, it might involve supervised lifting, carrying, or posture training. (b) Strategic Communication Coaching for Workplace Negotiation: Role-Playing: Patients participated in structured role-playing exercises with the nurse or rehabilitator acting as the employer. They practiced disclosing their health status in a positive frame, articulating their functional abilities rather than limitations.	In-person (hospital) transitioning to remote (post-discharge). Implementing personnel: Psychologists, public health specialists
Mezzo system	Family support aspect	1. Goal: To mobilize family support and transform it into effective actions that facilitate the patient’s RTW process, while mitigating family-related stressors. 2. Content: The intervention consisted of two integrated components: (1) Educational and Collaborative Problem-Solving Sessions: (a) Enhance Understanding: Educate them on the critical role of psychosocial support in achieving a full recovery and successful RTW, moving beyond viewing recovery as solely a physical process. (b) Foster Collaboration: Involve them in identifying and problem-solving specific RTW challenges faced by the patient (e.g., managing energy levels, adjusting daily routines). This process helped the family unit collaboratively reallocate household roles and responsibilities to reduce the patient’s burden and associated psychological stress. (2) Implementation of a Structured Family Support Checklist: This tool offered concrete, actionable recommendations across key domains, such as: Social Participation: Encouraging and accompanying the patient in gradual social and light physical activities to rebuild confidence. Emotional Support: Practicing active listening and positive reinforcement while avoiding overprotective behaviors that could undermine the patient’s self-efficacy.	In-person (hospital) transitioning to remote (post-discharge). Implementing personnel: Cardiology nurses
	Patient mutual support aspect	2. Goal: To leverage peer influence for psychosocial encouragement and practical learning. 2 Content: (1) The peer support component was delivered through structured sharing meetings, which served as a platform for facilitated knowledge exchange and psychosocial encouragement. (2) In practice, the research team proactively selects and invites CHD patients who have successfully RTW to participate in each meeting and share their personal experiences. These sharing sessions focus on core challenges such as managing physical limitations in the workplace, communication techniques with employers, and anxiety management. Following the structured sharing, an interactive discussion segment is held where participants can pose specific questions to peers and receive advice.	Online (Tencent Meeting).
Macro system	Policy and system aspect	1. Goal: To enhance awareness and utilization of relevant social security and healthcare policies to alleviate financial burdens. 2. Content: Provided information and assistance regarding: (1) Eligibility and application procedures for disability and medical subsidies in Wuxi; (2) Contacts for local insurance/human resources bureaus to facilitate independent inquiries	A single 30-min session pre-discharge Implementing personnel: Clinical nurses
	Concept aspect	1. Goal: To foster an accurate understanding of CHD and promote secondary prevention. 2. Content: Included: (1) A structured educational session using slideshows to explain CHD pathophysiology, treatment, and prognosis, correcting common misconceptions (e.g., over-reliance on stents); (2) A WeChat official account was established to regularly push health education materials to participants.	In-person (hospital) transitioning to remote (post-discharge). Implementing personnel: Cardiology nurses, physicians

### 2.7 Outcome measures and data collection

To comprehensively evaluate the effectiveness of the VR intervention, we assessed a range of outcomes that capture the multifaceted nature of returning to work. These measures include the following: RTW, RTW-SE, QoL and anxiety.

#### 2.7.1 RTW rate

In this study, RTW was operationally defined as either successfully resuming the patient’s previous employment or securing new paid employment within 3 months post-hospitalization (8). The RTW rate, calculated as the proportion of employed cases relative to the total sample size, was systematically tracked by research personnel through telephone or WeChat follow-ups conducted within the 3-month period following hospital discharge. We collected data on patients’ RTW status at two time points: 1 month after intervention initiation and at the end of the intervention period.

#### 2.7.2 RTW-se

It was assessed with the Chinese version of the RTW-SE questionnaire, which was culturally adapted by Liu (27). The questionnaire consists of 8 positive statements and 3 negative statements. Participants evaluated work-related statements using a 6-point Likert scale ranging from 1 (strongly disagree) to 6 (strongly agree). The final scale score is the mean score, which is calculated by summing up all of the entries. Higher scores indicate greater levels of RTW-SE. An earlier study with patients with CHD found that the Chinese version of the RTW-SE has strong validity and reliability, with a Cronbach’s *α* value of 0.882 (8). At baseline and at the end of the intervention, data were gathered.

#### 2.7.3 QoL

China questionnaire of quality of life in patients with cardiovascular diseases (28) was adopted to measure the QoL of patients. This instrument comprises 24 items organized into six domains: physical activity status (2 items: physical condition and participation in rehabilitation exercise), disease status (5 items: angina pectoris, palpitations, dyspnea, disease knowledge, and perspective on life and death), medical status (2 items: satisfaction with treatment and medical staff), general living status (5 items: diet, sleep, self-perception, recreation, and sexual life), psychosocial status (7 items: depression, anxiety, memory, intelligence, life confidence, relationships with relatives, and marital relationship), and employment status (2 items: work ability and interpersonal relationships at work). It employs a scoring system ranging from 0 to 154, where higher scores reflect better QoL. The questionnaire has a Cronbach’s *α* value of 0.910. Data were collected at baseline and at the end of the intervention.

#### 2.7.4 Anxiety

The Generalized Anxiety Disorder 7-item scale (GAD-7) was utilized to measure the anxiety of patients. Developed by Spitzer (29), the GAD-7 consists of seven items, each of which is assessed on a 4-point Likert scale (0–3). Higher total scores reflect more severe symptoms of anxiety. With a Cronbach’s *α* value of 0.800, when it used with CHD patients (30). Both at baseline and after the finish of the intervention, data were collected.

### 2.8 Adherence

Participatory diaries and communication logs were utilized to monitor patient adherence and engagement. A specialist researcher reviewed diary entries and interactions to track participation. Beyond mere adherence tracking, this method provided qualitative insights into the intervention’s process and immediate impact. For instance, the questions patients asked and the topics they raised in the WeChat group served as real-time indicators of their concerns, understanding of the intervention content, and the perceived relevance of the support provided. To further enhance adherence and engagement, we established a dedicated WeChat group where researchers answered questions, offered tailored health advice, and facilitated daily interactions. Additionally, participants received a personal phone call or WeChat message reminder prior to each intervention session.

### 2.9 Statistical analysis

Data were analyzed by an independent statistician using IBM SPSS Statistics Version 29 software. For continuous variables with non-normal distribution, data are presented as median (interquartile range). Comparisons between groups were made using the Mann–Whitney *U* test, and within-group (paired) comparisons were made using the Wilcoxon signed-rank test. The categorical data were presented as frequencies and proportions. The chi-square test was used to compare differences in count data between the two groups. A *p*-value of less than 0.05 was considered statistically significant, and all statistical tests were two-sided.

## 3 Results

The outcomes of the VR program are presented and interpreted through the lens of the SET, demonstrating its utility in explaining the intervention’s effects across different levels of the patient’s ecosystem. The results collectively illustrate a pathway of change, initiated at the individual level and supported by the social environment, leading to the successful reintegration of patients into the workforce.

### 3.1 Baseline data comparison

A total of 180 patients were recruited, from which 22 were excluded, remaining 158 eligible individuals were randomly assigned to the intervention or control group. At the end of the intervention, a total of 143 participants completed the entire trial (73 in the control group and 70 in the intervention group), and the data from all 143 individuals were included in the statistical analyses. Figure 1 shows the process of the study and follow-up. Between the two groups, there were no statistically significant differences in the baseline data (*p* > 0.05). As presented in Table 3.

**Table 3:** Baseline data comparison between the intervention and control group.

Variables	Control group (*n* = 73)	Intervention group (*n* = 70)	Test statistic value	*p* value
Age (Years)	55.00 (46.50,59.00)	57.00 (46.00,59.25)	−0.406^a^	0.685
Gender				
Male	61 (83.6)	60 (85.7)	0.127^b^	0.818
Female	12 (16.4)	10 (14.3)		
Marital status				
Unmarried	5 (6.8)	9 (12.8)	1.714^b^	0.424
Married	63 (86.3)	55 (78.6)		
Divorced or widowed	5 (6.9)	6 (8.6)		
Living arrangements				
Live alone	11 (15.1)	13 (18.6)	0.517^b^	0.772
Live with family	55 (75.3)	49 (70.0)		
Other	7 (9.6)	8 (11.4)		
Educational level				
Primary school or below	9 (12.3)	11 (15.7)	1.546^b^	0.672
Junior high school	27 (37.0)	21 (30.0)		
Senior high school	21 (28.8)	25 (35.7)		
College or above	16 (21.9)	13 (18.6)		
Monthly income (Yuan)				
<5,000	38 (52.1)	30 (42.9)	1.212^b^	0.271
≥5,000	35 (47.9)	40 (57.1)		
Hours worked per day before illness				
≤8	33 (45.2)	26 (37.1)	0.958^b^	0.328
>8	40 (54.8)	44 (62.9)		
Pre-illness work intensity				
Mild	11 (15.1)	13 (18.6)	1.986^b^	0.370
Moderate	33 (45.2)	37 (52.8)		
Severe	29 (39.7)	20 (28.6)		
NYHA Class				
I	22 (30.1)	21 (30.0)	0.502^b^	0.778
II	41 (56.2)	42 (60.0)		
III	10 (13.7)	7 (10.0)		
Number of stents				
0	13 (17.8)	18 (25.8)	1.594^b^	0.451
1	33 (45.2)	26 (37.1)		
≥2	27 (37.0)	26 (37.1)		
EF (%)	51.00 (50.00,56.00)	52.00 (50.00,56.00)	−0.477^a^	0.633
RTW-SE (Scores)	4.00 (3.50,5.00)	4.00 (4.00,5.00)	−0.219^a^	0.827
Total QoL (Scores)	69.00 (60.00,70.00)	67.50 (58.25,69.00)	−1.211^a^	0.226
Physical status (Scores)	18.00 (12.00,22.00)	18.00 (16.00,18.00)	−0.731^a^	0.465
Illness (Scores)	14.00 (11.50,16.00)	14.00 (13.00,15.00)	−0.209^a^	0.835
Medical conditions (Scores)	5.00 (4.00,5.00)	5.00 (4.00,6.00)	−1.097^a^	0.272
General living (Scores)	11.00 (10.00,13.00)	11.50 (10.00,12.00)	−0.752^a^	0.452
Social and psychological status (Scores)	12.00 (11.00,16.00)	14.00 (11.00,14.25)	−0.917^a^	0.359
Occupational status (Scores)	4.00 (3.00,5.00)	4.00 (4.00,5.00)	−0.530^a^	0.596
Anxiety (Scores)	5.00 (4.00,6.00)	5.00 (4.00,6.25)	−0.185^a^	0.853

^a^Z value; ^b^*χ*^2^ value. NYHA, New York Heart Association Functional; EF, Ejection fraction; RTW-SE, Return-to-work self-efficacy; QoL, Quality of Life.

### 3.2 Comparison of RTW-SE and anxiety scores

At the micro-system level, which encompasses the individual’s internal psychological state, the intervention group demonstrated a statistically significant improvement in RTW-SE scores compared to the control group. Concurrently, a marked reduction in anxiety scores was observed. As detailed in Table 4.

**Table 4:** Comparison of RTW-SE and anxiety scores between the two groups.

Variables	Time	Control group (*n* = 73)	Intervention group (*n* = 70)	*Z* value	*p* value
RTW-SE	Baseline	4.00 (3.50,5.00)	4.00 (4.00,5.00)	-0.219	0.827
	At the end of the intervention	4.00 (4.00,5.00)	5.00 (4.00,6.00)	−5.569	<0.001
	*Z* value	−1.117	−5.339	–	–
	*p* value	0.264	<0.001	–	–
Anxiety	Baseline	5.00 (4.00,6.00)	5.00 (4.00,6.25)	−0.185	0.853
	At the end of the intervention	5.00 (4.00,6.00)	4.00 (3.00,5.00)	−3.400	<0.001
	*Z* value	−1.920	−5.482	–	–
	*p* value	0.055	<0.001	–	–

### 3.3 Comparison of QoL scores

The significant enhancement in overall QoL reflects the interplay between micro- and mezzo-systems, stemming not only from physical recovery but also from strengthened psychosocial support at the mezzo-level. At the end of the intervention, the intervention group demonstrated significantly higher overall QoL scores and dimension-specific scores compared to the control group, as presented in Table 5. Wilcoxon signed-rank tests indicated that from baseline to post-intervention, scores significantly increased in the intervention group for all QoL measures. In the control group, significant increases were limited to total QoL, physical condition, medical condition, general living, and occupational status, with no significant change in illness or social-psychological status scores.

**Table 5:** Comparison of QoL between the two groups.

Variables	Time	Control group (*n* = 73)	Intervention group (*n* = 70)	*Z* value	*p* value
Total QoL	Baseline	69.00 (60.00,70.00)	67.50 (58.25,69.00)	−1.211	0.226
	At the end of the intervention	69.00 (65.50,77.00)	84.00 (70.00,91.00)	−5.864	<0.001
	*Z* value	−5.024	−6.488		
	*p* value	<0.001	<0.001		
Physical status	Baseline	18.00 (12.00,22.00)	18.00 (16.00,18.00)	−0.731	0.465
	At the end of the intervention	22.00 (18.00,22.00)	22.00 (20.00,32.00)	−1.211	0.226
	*Z* value	−5.062	−6.291		
	*p* value	<0.001	<0.001		
Illness	Baseline	14.00 (11.50,16.00)	14.00 (13.00,15.00)	−0.209	0.835
	At the end of the intervention	14.00 (11.00,16.00)	19.00 (17.00,21.00)	−7.820	<0.001
	*Z* value	−0.637	−6.499		
	*p* value	0.524	<0.001		
Medical conditions	Baseline	5.00 (4.00,5.00)	5.00 (4.00,6.00)	−1.097	0.272
	At the end of the intervention	5.00 (5.00,6.00)	6.00 (5.00,6.00)	−4.537	<0.001
	*Z* value	−2.481	−4.254		
	*p* value	0.013	<0.001		
General living	Baseline	11.00 (10.00,13.00)	11.50 (10.00,12.00)	−0.752	0.452
	At the end of the intervention	13.00 (12.00,13.00)	12.00 (10.00,13.00)	−3.030	0.002
	*Z* value	−4.738	−1.986		
	*p* value	<0.001	0.047		
Social-psychological status	Baseline	12.00 (11.00,16.00)	14.00 (11.00,14.25)	−0.917	0.359
	At the end of the intervention	13.00 (11.00,16.00)	18.00 (16.00,20.00)	−6.079	<0.001
	*Z* value	−1.273	−5.870		
	*p* value	0.203	<0.001		
Occupational status	Baseline	4.00 (3.00,5.00)	4.00 (4.00,5.00)	−0.530	0.596
	At the end of the intervention	5.00 (4.00,5.50)	5.00 (4.00,6.00)	−2.000	0.045
	*Z* value	−3.666	−3.886		
	*p* value	<0.001	<0.001		

### 3.4 Comparison of RTW rate

Successful RTW, a key indicator of social reintegration, is posited as the ultimate outcome of improvements in micro- and meso-level systems, and this was empirically demonstrated by a significantly higher rate of RTW in the intervention group compared to the control group. At the end of the intervention (3 months post-intervention), 45 participants returned to work in the control group and 61 participants in the intervention group, and the chi-square test was used to compare the difference in the rate of RTW between the two groups, and the results showed that the difference was statistically significant (*χ*^2^ = 12.114, *p* < 0.001). As shown in Table 6.

**Table 6:** Comparison of RTW rates between the two groups.

Return to work	Control group (*n* = 73)	Intervention group (*n* = 70)	*χ*^2^ value	*p* value
Yes	45(61.6)	61(87.1)	12.114	<0.001
No	28(38.4)	9(12.9)		

To identify factors associated with RTW and to isolate the effect of the intervention, a multivariable backward stepwise logistic regression was performed. The dependent variable was RTW status (0 = no, 1 = yes). Independent variables included group assignment (intervention = 1, control = 0), along with key demographic and clinically relevant factors as covariates. After adjustment, the model results robustly demonstrated that patients in the intervention group had 5.204 times the odds of successfully returning to work compared to those in the control group [95% CI (2.060, 13.148), *p* < 0.001]. Detailed results are presented in Table 7.

**Table 7:** After adjusting for potential confounding variables, a logistic regression analysis was conducted to examine employment status at 3 months post-intervention, stratified by VR intervention.

Variables	*B*	*Wald*	*P value*	*Exp(B)*	95% Confidence interval for *EXP(B)*	
					Lower limit	Upper limit
Group^①^ (Intervention group)	1.649	12.167	0.000	5.204	2.060	13.148
Age	−0.110	8.463	0.004	0.896	0.832	0.965
Educational Level^②^		7.593	0.055			
Junior high school	0.184	0.038	0.845	1.202	1.081	30.034
Senior high school	1.242	2.164	0.141	3.462	0.662	18.104
College or above	1.740	4.210	0.040	5.698	0.190	7.612

^①^Reference group: control group; ^②^Reference category: primary school or lower.

## 4 Discussion

As early as 2013, China issued the “Chinese Expert Consensus on Cardiac Rehabilitation and Secondary Prevention of Coronary Heart Disease” (31), which clearly identified VR as one of the five major components of cardiac rehabilitation for CHD. The ultimate goal of rehabilitation for patients with CHD is to help them reintegrate into their families and society. With the younger trend of CHD and the advancement of treatment methods, post-discharge patients are facing difficulties in reintegrating into society and returning to work. However, patients with CHD face multiple challenges in the process of returning to work, including physical function limitations, psychological adaptation disorders, and insufficient social support resources. These factors collectively lead to a persistently high rate of failure in returning to work. Based on the society ecosystems theory, our study innovatively constructed a multidimensional intervention program covering micro-(individual), meso- (family, patient mutual support) and macro- (policy, concepts) systems.

The results demonstrated significantly higher RTW rates in the intervention group (87.1%) compared to controls (61.6%) at 3-month follow-ups. The results of our previous research study confirmed that the RTW rate of young and middle-aged patients with CHD was 60.8% 3 months after discharge, and this intervention program significantly improved this indicator (25). This fully demonstrates that VR interventions designed based on society ecosystems theory can significantly enhance the success rate of RTW for CHD through multilevel systematic interventions. The following factors may contribute to the higher rate of patients returning to work following VR. First, according to the multidisciplinary teamwork model, VR programs integrate social support, psychological adjustment, vocational adjustment, and physical recovery. This comprehensive intervention approach successfully increases the patients’ capacity for occupational reintegration (32). Furthermore, research has demonstrated that good psychosocial assistance significantly lowers barriers to returning to the workforce (33, 34). We have established an integrated hybrid support program that provides patients with timely professional guidance through WeChat groups, while regularly organizing peer support activities to facilitate experience sharing and emotional support. This comprehensive support system not only enhances patients’ treatment adherence, but also creates favorable conditions for their RTW by alleviating psychological stress and strengthening their sense of social belonging.

RTW-SE reflects patients’ confidence in their ability to resume work and is a strong predictor of successful return to employment (35). The results of this study showed that before the intervention, the RTW-SE scores of the intervention and the control group were (4.09 ± 0.86) and (4.07 ± 0.89), respectively, which were generally consistent with previous studies (8, 36). The intervention group’s RTW-SE scores were significantly higher than the control group’s at the end of the intervention. Possible reasons for this may be as follows: the intervention program of this study was formulated on the basis of in-depth understanding and analysis of the occupational rehabilitation needs of patients with CHD through qualitative interviews, and targeted intervention measures were taken to accurately enhance patients’ confidence in returning to work. In terms of intervention forms, based on the patients’ VR needs, this study adopted a diversified intervention approach, providing VR guidance to patients through channels such as WeChat groups, WeChat official accounts, Tencent Meetings, and telephone follow-ups. In addition, the experience sharing of patients who have successfully worked can enable patients to learn from others’ successful experiences in getting back to work, so that they can have confidence in re-entering the workforce through the “role modeling” effect (37). The support and encouragement from family members further enhanced the patients’ vocational self-efficacy. This implies that digital platforms and peer modeling can be fully utilized in work rehabilitation interventions to help patients build up their vocational confidence through multi-channel support and facilitate their successful job return.

At the baseline assessment, the total QoL scores for the intervention group and control group were (63.79 ± 9.11) and (64.23 ± 0.72), respectively, which were slightly higher than the findings reported in a local study (38). It was found that the possible reasons for this difference were: the subjects of this study were selected from the tertiary hospitals in the Wuxi area, and the economic level of the Wuxi area is more highly developed compared with that of the Huai’an area. Moreover, the proportion of patients with NYHA class III heart failure in our study (11.89%) was significantly lower than that reported in the reference study (17.3%). These collective factors may explain the superior QoL scores observed in our study.

Following the 3-month intervention, the SET-based VR program demonstrated a profound and multifaceted impact, evidenced by the significantly higher total QoL in the intervention group compared to the control group. This enhancement can be deconstructed through the lens of the SET framework, which guided the intervention’s multi-level design: First, at the macro-system level, the intervention program actively guided patients in applying for disease subsidies and assisted them in adjusting family roles, which effectively alleviated both financial burdens and psychological stress (39, 40). This systemic support provided a more stable foundation upon which other rehabilitative efforts could build. Concurrently, at the micro-system level, the intervention targeted the individual’s physical and psychological capacity. The delivery of risk-stratified, individualized exercise prescriptions led directly to significant improvements in physical status. In addition, the provision of comprehensive disease knowledge empowered patients by strengthening their coping abilities and reducing illness-related discomfort. Importantly, specific components including facilitated sessions, support journaling, and structured education were designed to build RTW-SE and manage anxiety. These components were instrumental in driving the notable gains in socio-psychological status (17.77 ± 3.28 vs. 13.71 ± 3.55, *p* < 0.001). This integrated focus on both physical and psychological well-being constituted a central pathway through which QoL was enhanced (41). Finally, at the mezzo-system level, the intervention mobilized the patient’s immediate social environment to foster sustained support. The organized peer support sessions, featuring structured narratives from successful returners, and the family role adjustment activities, facilitated by a practical support checklist, created a reinforcing network of emotional and practical aid. These activities played a pivotal role in enhancing patients’ perceptions of social and family support. This improvement, in turn, significantly contributed to their social-psychological well-being and overall life satisfaction (42, 43). In conclusion, the comprehensive improvement in QoL observed in this study stems from the synergistic effect of a multi-level intervention guided by the SET framework. By simultaneously addressing macro-level financial barriers, rebuilding micro-level physical and psychological capacity, and activating mezzo-level social networks, this approach demonstrates the necessity of moving beyond singular-focus interventions in clinical practice. Providing timely, multidimensional support is thus essential to facilitating role transition and achieving holistic QoL improvement in CHD recovery.

Anxiety is closely associated with RTW (44, 45), and RTW itself may alleviate anxiety symptoms, the functional impairment from CHD and pre-existing anxiety can conversely delay successful occupational reintegration (37, 46). In this study, a 3-month job rehabilitation intervention demonstrated significant improvements in patients’ anxiety levels, attributable to its synergistic multi-system effects across microsystem, mesosystem, and macrosystem. At the microsystem level, individuals enhance their physical well-being through consistent exercise, alleviate concerns stemming from bodily discomfort, and facilitate adaptation to occupational transitions (10, 47). This process involves balancing work demands with health requirements and mitigating anxiety related to job performance through bolstering individual capabilities and self-assurance. At the mesosystemic level, our research assisted patients in redefining their roles and responsibilities, establishing structured family support, enhancing peer support via online peer contact, and giving patients bravery and warmth through a strong social support system to lessen stress and anxiety (48). At the macrosystem level, we assisted patients in learning about and gaining access to pertinent resources through policy navigation to reduce financial strains. We also assisted patients to acquire a correct understanding of the condition in order to reduce excessive anxiety brought on by financial strains and misunderstandings about it from the standpoint of useful problem-solving and cognitive enhancement. For healthcare professionals, it is recommended to integrate targeted psychological guidance modules into work rehabilitation interventions so as to strengthen the effect of the intervention through the dual path of “problem-solving + emotion regulation.” At the same time, it is recommended to establish a dynamic evaluation mechanism for work rehabilitation interventions so that intervention strategies can be adjusted in a timely manner to continuously optimize the effectiveness of anxiety improvement.

After adjusting for confounders, logistic regression confirmed a strong positive effect of VR intervention on RTW (Adjusted OR = 5.204, *p* < 0.001). This main effect remained robust after controlling for baseline factors. Specifically, while higher education facilitated and older age hindered RTW, gender and living arrangements were not significant confounders, suggesting the intervention’s benefits may be broadly applicable. This finding holds significant clinical and practical implications. It demonstrates that VR is not merely an optional “add-on” service in cardiac care, but rather an essential component of comprehensive rehabilitation, playing a critical role in enhancing the return-to-work rate and QoL for working-age patients.

The findings of this study underscore the critical importance of personalized assessment as a foundation for effective vocational rehabilitation in young and middle-aged patients with coronary heart disease. The substantial improvements observed in return-to-work rates, return-to-work self-efficacy, quality of life, and anxiety appear to be closely linked to the individualized assessment of patients’ physical capacity, psychological readiness, occupational demands, and contextual barriers prior to and during the intervention. By systematically mapping individual job characteristics, functional limitations, perceived barriers, and available social resources, the intervention was able to align rehabilitation strategies with patients’ real-life work situations and recovery trajectories. These results suggest that personalized assessment is not merely a preparatory step, but a key mechanism through which rehabilitation interventions can effectively translate clinical recovery into sustainable work participation.

Several limitations should be acknowledged in this study. First, as a single-center trial conducted at a tertiary hospital in Wuxi, its generalizability may be limited, and future multi-center studies are needed to validate the effectiveness of VR. Second, this study did not incorporate the perspectives of employers and workplaces. Subsequent research should integrate these factors to facilitate the development of more comprehensive and effective RTW strategies for working-age patients. Finally, the duration of the VR intervention was relatively short, which may have restricted the magnitude of the effects we observed. Due to the lack of long-term follow-up data after the intervention ended, we cannot determine the sustainability of the improvements in employment status. Despite these limitations, we believe our findings provide valuable preliminary evidence for the efficacy of VR.

## 5 Conclusion

In this study, the interventions were developed from multiple perspectives based on the social-ecological system theory. Through a 3-month VR intervention for young and middle-aged patients with CHD, the patients’ RTW rate (the main objective) was significantly improved, and the patients’ RTW-SE, QoL were also enhanced. This study innovatively applied the SET to the field of VR for CHD, and established a generalizable intervention framework that takes into account biomedical, psychosocial, and environmental factors. Follow-up studies could focus on long-term outcome tracking and optimization of differentiated programs for different occupational groups.

## Data Availability

The raw data supporting the conclusions of this article will be made available by the authors, without undue reservation.
